# An Entropy-Weighted Multi-Factor Ambiguity Subset Selection Algorithm for Partial Ambiguity Resolution in Multi-GNSS and Multi-Frequency Precise Point Positioning

**DOI:** 10.3390/s26144388

**Published:** 2026-07-10

**Authors:** Mingduan Zhou, Lu Qin, Likun Cui, Qiao Song, Shiqi Lin, Peng Yan, Shufa Li, Qianlong Xie, Yuhan Qin, Zihan Zhou, Guanxiu Wu

**Affiliations:** 1 School of Geomatics and Urban Spatial Informatics, Beijing University of Civil Engineering and Architecture, Beijing 102616, China; 2108160124002@stu.bucea.edu.cn (Q.S.); 2108570424086@stu.bucea.edu.cn (S.L.); 2108160125006@stu.bucea.edu.cn (P.Y.); 2108570425026@stu.bucea.edu.cn (S.L.); 2108160123008@stu.bucea.edu.cn (Q.X.); 2108570023128@stu.bucea.edu.cn (Y.Q.); 2108160225013@stu.bucea.edu.cn (Z.Z.); 2108160225010@stu.bucea.edu.cn (G.W.); 2 Shanghai Surveying and Mapping Institute, Shanghai 200063, China; sjiao2050350990@gmail.com

**Keywords:** multi-GNSS and multi-frequency PPP, undifferenced and uncombined PPP, partial ambiguity resolution, entropy-based adaptive weighting, multi-factor ranking and screening, ambiguity subset selection

## Abstract

**Highlights:**

**What are the main findings?**

- An entropy-weighted multi-factor ambiguity subset selection algorithm is proposed for partial ambiguity resolution. The proposed MPAR algorithm integrates SNR, ambiguity variance, and carrier-phase residuals for reliable subset selection.
- MPAR achieves ambiguity-fixing rates of 98.9%, 98.7%, and 99.2% under five-, four-, and three-frequency con-figurations. MPAR increases the WL and NL residual proportions within ±0.1 cycle by 13.6% and 46.4% over VAR, respectively.

**What are the implications of the main findings?**

- The proposed algorithm improves ambiguity subset reliability in high-dimensional multi-GNSS and multi-frequency PPP.
- Entropy-based adaptive weighting provides a flexible alternative to single-factor or fixed-weight PAR strategies.

**Abstract:**

Reliable ambiguity subset selection is essential for partial ambiguity resolution (PAR) in multi-GNSS and multi-frequency precise point positioning (PPP), as the increasing number of satellite–frequency ambiguities expands the ambiguity search space and reduces ambiguity-fixing reliability in high-dimensional scenarios. To address this issue, this study proposes a multi-factor ranking and screening partial ambiguity resolution (MPAR) algorithm, an entropy-weighted multi-factor ambiguity subset selection algorithm designed for multi-GNSS and multi-frequency undifferenced and uncombined precise point positioning (UDUC PPP). The proposed MPAR algorithm evaluates candidate ambiguities using three quality indicators: signal-to-noise ratio, ambiguity variance, and carrier-phase residual. Min–max normalization is used to eliminate scale differences among the indicators, while entropy-based adaptive weighting is introduced to dynamically determine their relative contributions. Based on the integrated ranking results, ambiguities are divided into easy-to-fix and hard-to-fix subsets, with the hard-to-fix subset further refined through iterative screening before integer fixing. The proposed algorithm was validated using 24 h BDS-3/GPS/Galileo observations collected from 11 globally distributed MGEX stations on day 350 of 2025 under five-, four-, and three-frequency configurations. Its performance was compared with the baseline full ambiguity resolution strategy (FAR), which fixes all candidate ambiguities without subsequent iterative exclusion after an initial fixing failure, as well as elevation-angle-factor-based partial ambiguity resolution (ELE) and variance-factor-based partial ambiguity resolution (VAR). The MPAR algorithm achieved ambiguity-fixing rates of 98.9%, 98.7%, and 99.2% under the three configurations, respectively, exhibiting performance comparable to ELE while outperforming VAR and FAR. Compared with VAR, MPAR increased the average proportions of wide-lane and narrow-lane ambiguity residuals within ±0.1 cycle by 13.6% and 46.4%, respectively. Under the five-frequency configuration, MPAR achieved the best overall performance, with horizontal and vertical convergence times of 9.1 and 8.1 min, respectively. These results demonstrate that the proposed entropy-weighted multi-factor subset selection algorithm improves ambiguity estimation quality and enhances the reliability and convergence performance of high-dimensional multi-GNSS and multi-frequency PPP.

## 1. Introduction

In multi-GNSS and multi-frequency precise point positioning (PPP), the number of ambiguity parameters increases substantially as additional frequencies and GNSS constellations are incorporated into the estimation process. The resulting increase in ambiguity dimensionality enlarges the integer ambiguity search space, thereby increasing the computational burden and reducing ambiguity-fixing efficiency and reliability. In high-dimensional ambiguity resolution, direct full ambiguity resolution is often computationally demanding and more susceptible to fixing failure or incorrect fixing [1]. In practical GNSS environments, observations are inevitably affected by various error sources, including multipath effects, ionospheric delay, tropospheric delay, and measurement noise. Ambiguities associated with satellites of poor observation quality generally have lower estimation accuracy and reliability. Directly including these low-quality ambiguities in the fixing process may increase the risk of incorrect fixing and degrade PPP accuracy and stability. Therefore, partial ambiguity resolution (PAR), which fixes only reliable ambiguity subsets, provides an effective strategy for improving ambiguity-fixing reliability in multi-GNSS and multi-frequency PPP.

The key to PAR is the selection of reliable ambiguity subsets. Efficiently selecting high-precision and reliable ambiguity subsets from high-dimensional ambiguity parameters is critical for improving ambiguity resolution performance in multi-GNSS and multi-frequency PPP. Parkins et al. used the satellite signal-to-noise ratio (SNR) as a selection criterion and excluded satellites with low SNR before ambiguity fixing [2]. Teunissen selected ambiguity subsets based on bootstrapping success rates and iteratively excluded ambiguities with the lowest success rates until a predefined threshold was satisfied. Similar strategies were subsequently investigated by Nardo et al. [3,4]. Mowlam and Dutta ranked ambiguities based on satellite elevation angles and iteratively removed ambiguities associated with low-elevation satellites to improve ambiguity-fixing performance [5,6]. Dai proposed removing ambiguities whose integer values were inconsistent between the best and second-best candidates after full ambiguity resolution failed the ratio test, using candidate inconsistency as the sole selection criterion [7]. Zhang et al. selected ambiguity subsets based on ambiguity variance and iteratively removed the wide-lane ambiguity with the largest variance, followed by repeated LAMBDA search and ratio-test validation until successful fixing was achieved [8]. Wen et al. selected ambiguity subsets by minimizing the trace of the variance–covariance matrix of the fixed solution while constraining float ambiguities to remain stable within a temporal window [9]. Brack proposed a generalized integer aperture estimation method in which ambiguity elements within the full ambiguity vector were individually tested, and only those failing the consistency test were excluded. In subsequent work, the integer least-squares failure rate was further used as a subset selection criterion [10,11]. Psychas et al. used the ambiguity success rate (ASR) derived from the LAMBDA method as the primary criterion for PAR subset selection and systematically analyzed the influence of multi-GNSS and multi-frequency observations on PAR performance [12]. Castro-Arvizu et al. selected ambiguity subsets based on the formal precision of potential fixed solutions and iteratively reduced the subset size until the trace of the variance–covariance matrix satisfied a predefined threshold [13]. Zhang et al. combined ambiguity dilution of precision (ADOP) with optimal stopping theory to dynamically determine the optimal subset size without predefined thresholds, thereby balancing the number of ambiguities and the fixing success rate [14]. Henkel et al. used the upper bound of conditional ambiguity bias as the sole criterion to control the integer decorrelation process, retaining only ambiguities that satisfied the bias constraint for fixing while treating the remaining ambiguities as float solutions [15]. Although these PAR methods have achieved promising performance, most rely on a single selection criterion for ambiguity subset determination, which limits their adaptability and robustness in high-dimensional PPP scenarios.

To improve the robustness and reliability of PAR methods in ambiguity subset determination, multiple selection criteria have been jointly introduced. Yan et al. used Schmidt orthogonalization for reference satellite selection and combined the ratio test with Bayesian posterior probability for narrow-lane ambiguity subset determination [16]. Wang et al. jointly used bootstrapping success rates and ratio-test statistics as ambiguity validation criteria, fixing ambiguity subsets only when both thresholds were satisfied [17]. Hou et al. ranked decorrelated ambiguities and combined bootstrapping success rates with the fixed failure-rate ratio test (FFRT) for ambiguity-fixing validation [18]. In addition to validation statistics, ambiguity precision, observation geometry, and observation quality have also been incorporated into the ambiguity subset selection criteria. Yue et al. proposed a subset selection strategy that jointly considered float ambiguity variance, ambiguity dilution of precision (ADOP), position dilution of precision (PDOP), and ratio-test values, achieving better positioning performance than the conventional variance-based ranking method [19]. Zhao et al. proposed a two-stage ambiguity subset selection strategy in which ambiguities were first screened using satellite elevation angles and the IGG-III robust weight function, which is mainly used to reduce the influence of gross errors or outlying observations on parameter estimation, and were then ranked based on posterior observation weights and bootstrapping success rates [20]. Li et al. developed a sequential ambiguity selection strategy based on pseudorange and carrier-phase residuals, satellite elevation angles, and decorrelated ambiguity variances [21]. Li et al. further developed PAR algorithms based on ratio-test statistics, bootstrapping success rates, and ADOP by iteratively excluding satellites that caused the largest variations in validation metrics [22]. Sun et al. jointly used posterior observation weights and bootstrapping success rates for ambiguity subset determination [23].

Meanwhile, several studies have selected ambiguity subsets by ranking candidate ambiguities using weighted multi-factor scores or by applying multi-factor preset thresholds. Li et al. jointly used satellite elevation angle, signal-to-noise ratio, and float ambiguity variance for ambiguity subset selection, and Zhong et al. further introduced IGG-III robust preprocessing within this framework to improve ambiguity screening performance [24,25]. Dong et al. selected ambiguity subsets using satellite elevation angles and carrier-to-noise ratios through a weighted scoring function [26]. Zhu et al. generated extra-wide-lane and wide-lane ambiguities through parameter recombination and subsequently applied multiple constraint tests and epoch-differenced validation for narrow-lane ambiguity subset selection [27]. Peng et al. first excluded low-quality satellites based on elevation angles and signal-to-noise ratios and then removed ambiguities with the largest variances according to success-rate estimation until predefined thresholds were satisfied [28]. Ye et al. combined multiple PAR strategies, including time-differenced carrier phase (TDCP), elevation-angle-based, signal-to-noise-ratio-based, and decorrelated-variance-based methods, through parallel execution and result fusion [29]. Nevertheless, most existing multi-criteria PAR methods still rely on fixed weighting schemes or simple weighted combinations, which limits their ability to dynamically adjust the relative contributions of different indicators during ambiguity subset selection.

To address these limitations, this study proposes an entropy-weighted multi-factor ambiguity subset selection algorithm for partial ambiguity resolution in multi-GNSS and multi-frequency PPP, termed the multi-factor ranking and screening partial ambiguity resolution (MPAR) algorithm. The proposed MPAR algorithm is designed to identify reliable ambiguity subsets before integer fixing within an undifferenced and uncombined PPP framework. It adopts a two-stage ambiguity screening strategy that combines multi-factor ranking, adaptive weight determination, and iterative subset refinement. Specifically, signal-to-noise ratio, ambiguity variance, and carrier-phase residuals are jointly used as quality indicators to evaluate candidate ambiguities. Min–max normalization is applied to eliminate differences in scale and units among these indicators, and information entropy is introduced to dynamically determine their relative weights. In this way, an adaptive multi-factor ambiguity ranking and screening framework is established. Experimental validation was conducted using BDS-3/GPS/Galileo observations from 11 globally distributed MGEX stations under five-, four-, and three-frequency configurations. Ambiguity-fixing rate, wide-lane and narrow-lane residual distributions, convergence time, and positioning accuracy were systematically evaluated under different ambiguity resolution strategies.

The remainder of this paper is organized as follows. Section 2 presents the proposed methodology. Section 3 describes the experimental data and processing strategies. Section 4 presents the experimental results and discussion. Finally, Section 5 provides the conclusions and future research directions.

## 2. Proposed Method

**Figure 1:**
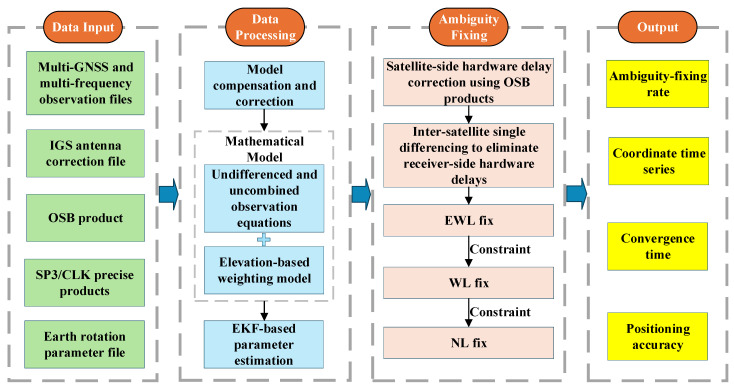
Overall Processing Flow of the Proposed Method.

In this study, multi-GNSS and multi-frequency precise point positioning (PPP) is implemented using an undifferenced and uncombined observation model combined with cascaded ambiguity resolution. The undifferenced and uncombined model preserves the original information of all observations and enables unified treatment of pseudorange and carrier-phase hardware delays at both the satellite and receiver sides, thereby allowing flexible integration of multiple GNSS constellations and frequencies [30]. The cascaded ambiguity resolution strategy sequentially fixes extra-wide-lane, wide-lane, and narrow-lane ambiguities, progressively reducing the ambiguity search space and improving ambiguity-fixing efficiency and reliability [31]. On this basis, the proposed MPAR algorithm focuses on reliable ambiguity subset selection for partial ambiguity resolution by adaptively ranking and screening candidate ambiguities. The resulting framework avoids noise amplification caused by ionosphere-free combinations while enabling efficient and reliable ambiguity fixing in high-dimensional multi-GNSS and multi-frequency PPP. The overall processing flow is shown in Figure 1.

### 2.1. Multi-GNSS and Multi-Frequency Undifferenced and Uncombined PPP Model

In multi-GNSS and multi-frequency PPP applications, after correcting for antenna phase center offset, relativistic effects, tidal effects, phase wind-up, and the dry component of tropospheric delay, the observation equations for pseudorange and carrier-phase measurements can be expressed as follows [32,33,34]:

(1) $$P_{r,j}^{s,Q}=\rho_{r}^{s,Q}+c\cdot dt_{r}^{Q}-c\cdot dt^{s,Q}+Mw_{r}^{s,Q}\cdot ZWD_{r}+\gamma_{1j}^{Q}\cdot I_{r,1}^{s,Q}+d_{r,P_{j}}^{Q}-d_{P_{j}}^{s,Q}+\varepsilon_{r,j}^{s,Q}$$

(2) $$L_{r,j}^{s,Q}=\rho_{r}^{s,Q}+c\cdot dt_{r}^{Q}-c\cdot dt^{s,Q}+Mw_{r}^{s,Q}\cdot ZWD_{r}-\gamma_{1j}^{Q}\cdot I_{r,1}^{s,Q}+\lambda_{j}^{Q}\cdot N_{r,j}^{s,Q}+b_{r,L_{j}}^{Q}-b_{L_{j}}^{s,Q}+\xi_{r,j}^{s,Q}$$

where $P_{r,j}^{s,Q}$ and $L_{r,j}^{s,Q}$ denote the corrected pseudorange and carrier-phase observations after model compensation, respectively; superscript *s* represents the satellite PRN number, and superscript *Q* denotes the GNSS constellation; subscripts r and j represent the receiver and frequency index, respectively; $\rho_{r}^{s,Q}$ denotes the geometric distance between satellite s and receiver r; $\lambda_{j}^{Q}$ represents the wavelength corresponding to frequency j; c is the speed of light in vacuum; $dt^{s,Q}$ and $dt_{r}^{Q}$ denote the satellite and receiver clock offsets, respectively; $Mw_{r}^{s,Q}$ represents the wet mapping function associated with satellite elevation angle; $ZWD_{r}$ denotes the zenith wet tropospheric delay; $\gamma_{1j}^{Q}={\left(f_{1}^{Q}\right)}^{2}/{\left(f_{j}^{Q}\right)}^{2}$ represents the frequency-dependent ionospheric amplification factor; $I_{r,1}^{s,Q}$ denotes the ionospheric delay corresponding to the first frequency $f_{1}^{Q}$; $N_{r,j}^{s,Q}$ represents the ambiguity parameter associated with the carrier-phase observation; $d_{P_{j}}^{s,Q}$ and $d_{r,P_{j}}^{Q}$ denote the pseudorange hardware delays at the satellite and receiver sides, respectively; $b_{L_{j}}^{s,Q}$ and $b_{r,L_{j}}^{Q}$ represent the carrier-phase hardware delays at the satellite and receiver sides, respectively; and $\varepsilon_{r,j}^{s,Q}$ and $\xi_{r,j}^{s,Q}$ denote the measurement noises of pseudorange and carrier-phase observations, respectively, including residual unmodeled errors.

Pseudorange hardware delays are generally regarded as relatively stable. For the pseudorange hardware delay biases associated with the first and second frequencies, the delays can generally be decomposed into frequency-independent and frequency-dependent components [35,36]. Accordingly, the satellite-side pseudorange hardware delay can be expressed as follows:

(3) $$\left.\begin{array}{l} d_{P_{1}}^{s,Q}=l^{s}+k^{s}\\ d_{P_{2}}^{s,Q}=l^{s}+\gamma_{1j}^{Q}\cdot k^{s} \end{array}\right\}\Rightarrow \left\{\begin{array}{l} l^{s}=d_{IF_{12}}^{s,Q}\\ k^{s}=\beta_{12}\cdot DCB_{12}^{s,Q} \end{array}\right.$$

Similarly, the receiver-side pseudorange hardware delay can be expressed as follows:

(4) $$\left.\begin{array}{l} d_{r,P_{1}}^{Q}=l_{r}+k_{r}\\ d_{r,P_{2}}^{Q}=l_{r}+\gamma_{12}^{Q}\cdot k_{r} \end{array}\right\}\Rightarrow \left\{\begin{array}{l} l_{r}=d_{r,IF_{12}}^{Q}\\ k_{r}=\beta_{12}\cdot DCB_{r,12}^{Q} \end{array}\right.$$

where $l^{s}$ and $k^{s}$ denote the frequency-independent and frequency-dependent components of the satellite-side pseudorange hardware delay, respectively. Among them, $l^{s}$ is absorbed into the satellite clock offset, while $k^{s}$ is corrected using DCB. Similarly, $l_{r}$ and $k_{r}$ represent the frequency-independent and frequency-dependent components of the receiver-side pseudorange hardware delay, respectively, where $l_{r}$ is absorbed into the receiver clock offset and $k_{r}$ is absorbed into the ionospheric delay parameter.

Carrier-phase hardware delays exhibit significant time-varying characteristics and are generally decomposed into constant and time-varying components. Accordingly, the satellite-side carrier-phase hardware delay $b_{L_{j}}^{s,Q}$ can be decomposed as follows:

(5) $$b_{L_{j}}^{s,Q}=\Delta b_{L_{j}}^{s,Q}+\delta b_{L_{j}}^{s,Q}$$

Similarly, the receiver-side carrier-phase hardware delay $b_{r,L_{j}}^{Q}$ can be decomposed as follows:

(6) $$b_{r,L_{j}}^{Q}=\Delta b_{r,L_{j}}^{Q}+\delta b_{r,L_{j}}^{Q}$$

where $\Delta b_{L_{j}}^{s,Q}$ and $\Delta b_{r,L_{j}}^{Q}$ denote the constant components of the satellite-side and receiver-side carrier-phase hardware delays, respectively. These components are assumed to remain constant and are absorbed into the integer ambiguity parameters. $\delta b_{L_{j}}^{s,Q}$ and $\delta b_{r,L_{j}}^{Q}$ represent the time-varying components of the satellite-side and receiver-side carrier-phase hardware delays, respectively, which are generally decomposed into frequency-independent and frequency-dependent terms. The time-varying component of the satellite-side carrier-phase hardware delay can be further expressed as follows:

(7) $$\left.\begin{array}{c} \delta b_{L_{1}}^{s,Q}=h^{s}-e^{s}\\ \delta b_{L_{2}}^{s,Q}=h^{s}-\gamma_{j}^{Q}\cdot e^{s} \end{array}\right\}\Rightarrow \left\{\begin{array}{c} h^{s}=\delta b_{IF_{12}}^{s,Q}\\ e^{s}=-\beta_{12}\cdot \delta DPB_{12}^{s,Q} \end{array}\right.$$

Similarly, the time-varying component of the receiver-side carrier-phase hardware delay can be further expressed as follows:

(8) $$\left.\begin{array}{c} \delta b_{r,L_{1}}^{Q}=h_{r}-e_{r}\\ \delta b_{r,L_{2}}^{Q}=h_{r}-\gamma_{j}^{Q}\cdot e_{r} \end{array}\right\}\Rightarrow \left\{\begin{array}{c} h_{r}=\delta b_{r,IF_{12}}^{Q}\\ e_{r}=-\beta_{12}\cdot \delta DPB_{r,12}^{\;\;Q} \end{array}\right.$$

where $h^{s}$ and $e^{s}$ denote the frequency-independent and frequency-dependent components of the time-varying satellite-side carrier-phase hardware delay, respectively. Among them, $h^{s}$ is absorbed into the satellite clock offset, while $e^{s}$ is absorbed into the ionospheric delay parameter. Similarly, $h_{r}$ and $e_{r}$ represent the frequency-independent and frequency-dependent components of the time-varying receiver-side carrier-phase hardware delay, respectively, where $h_{r}$ is absorbed into the receiver clock offset and $e_{r}$ is absorbed into the ionospheric delay parameter. It should be noted that this absorption is a parameter reparameterization in the undifferenced and uncombined PPP model. The estimated ionospheric delay parameter therefore contains the actual slant ionospheric delay together with the receiver-side frequency-dependent hardware term. No external dual-frequency or multi-frequency ionospheric correction is applied in the subsequent processing; therefore, the receiver-side frequency-dependent term is not corrected repeatedly.

The following variables can be defined:

(9) $$\left\{\begin{array}{l} DCB_{ij}^{s,Q}=d_{P_{1}}^{s,Q}-d_{P_{j}}^{s,Q}\\ DCB_{r,ij}^{Q}=d_{r,P_{1}}^{Q}-d_{r,P_{j}}^{Q}\\ \delta DPB_{ij}^{s,Q}=\delta b_{L_{1}}^{s,Q}-\delta b_{L_{j}}^{s,Q}\\ \delta DPB_{r,ij}^{Q}=\delta b_{r,L_{1}}^{Q}-\delta b_{r,L_{j}}^{Q}\\ \alpha_{1j}=\gamma_{ij}^{Q}/\left(\gamma_{ij}^{Q}-1\right)=f_{i}^{2}/\left(f_{i}^{2}-f_{j}^{2}\right)\\ \beta_{1j}=-1/\left(\gamma_{ij}^{Q}-1\right)=-f_{j}^{2}/\left(f_{i}^{2}-f_{j}^{2}\right)\\ d_{{\mathrm{IF}}_{ij}}^{s,Q}=\alpha_{ij}\cdot d_{P_{i}}^{s,Q}+\beta_{ij}\cdot d_{P_{j}}^{s,Q}\\ d_{r,{\mathrm{IF}}_{ij}}^{Q}=\alpha_{ij}\cdot d_{r,P_{i}}^{Q}+\beta_{ij}\cdot d_{r,P_{j}}^{Q}\\ \delta b_{{\mathrm{IF}}_{ij}}^{s,Q}=\alpha_{ij}\cdot \delta b_{L_{i}}^{s,Q}+\beta_{1j}\cdot \delta b_{L_{j}}^{s,Q}\\ \delta b_{r,{\mathrm{IF}}_{ij}}^{Q}=\alpha_{ij}\cdot \delta b_{r,L_{i}}^{Q}+\beta_{ij}\cdot \delta b_{r,L_{j}}^{Q} \end{array}\right.$$

where subscripts i and j denote the i-th and j-th frequencies, respectively; $DCB_{ij}^{s,Q}$ and $DCB_{r,ij}^{Q}$ represent the differential code biases (DCBs) at the satellite and receiver sides, respectively; $\delta DPB_{ij}^{s,Q}$ and $\delta DPB_{r,ij}^{Q}$ denote the differential time-varying phase biases at the satellite and receiver sides, respectively; $\alpha_{ij}$ and $\beta_{ij}$ represent the ionosphere-free combination coefficients, respectively; $d_{IF_{ij}}^{s,Q}$ and $d_{r,{\mathrm{IF}}_{ij}}^{Q}$ denote the satellite-side and receiver-side pseudorange hardware delays in the ionosphere-free combination, respectively; and $\delta b_{{\mathrm{IF}}_{ij}}^{s,Q}$ and $\delta b_{r,{\mathrm{IF}}_{ij}}^{Q}$ represent the time-varying components of the satellite-side and receiver-side carrier-phase hardware delays in the ionosphere-free combination, respectively.

Considering that precise satellite clock products are generally generated based on dual-frequency ionosphere-free combinations, the precise satellite clock offset $dt_{{\mathrm{IF}}_{12}}^{s,Q}$ contains both the pseudorange hardware delay and the time-varying carrier-phase hardware delay associated with the ionosphere-free combination, i.e.,

(10) $$c\cdot dt_{{\mathrm{IF}}_{12}}^{s,Q}=c\cdot dt^{s,Q}-d_{{\mathrm{IF}}_{12}}^{s,Q}-\delta b_{{\mathrm{IF}}_{12}}^{s,Q}$$

Let the following variables be defined:

(11) $$\left\{\begin{array}{l} \Delta {\bar{P}}_{r,1}^{s,Q}=P_{r,1}^{s,Q}+\beta_{12}\cdot DCB_{12}^{s,Q}-\rho_{r0}^{s,Q},\Delta {\bar{L}}_{r,1}^{s,Q}=L_{r,j}^{s,Q}-\rho_{r0}^{s,Q}\\ \Delta {\bar{P}}_{r,2}^{s,Q}=P_{r,2}^{s,Q}-\alpha_{1j}\cdot DCB_{12}^{s,Q}-\rho_{r0}^{s,Q}\\ \Delta {\bar{L}}_{r,2}^{s,Q}=L_{r,2}^{s,Q}-\rho_{r0}^{s,Q}\\ \Delta {\bar{P}}_{r,j}^{s,Q}=P_{r,j}^{s,Q}-\alpha_{12}\cdot DCB_{1j}^{s,Q}+\beta_{12}\cdot DCB_{2j}^{s,Q}-\rho_{r0}^{s,Q}\\ c\cdot d{\bar{t}}_{r}^{Q}=c\cdot dt_{r}^{Q}+d_{r,{\mathrm{IF}}_{12}}^{Q}+\delta b_{r,{\mathrm{IF}}_{12}}^{Q}\\ {\bar{I}}_{r,1}^{s,Q}=I_{r,1}^{s,Q}+\beta_{12}\cdot DCB_{r,12}^{Q}+\beta_{12}\cdot (\delta DPB_{r,12}^{Q}-\delta DPB_{12}^{s,Q})\\ \lambda_{j}^{Q}\cdot {\bar{N}}_{r,j}^{s,Q}=\lambda_{j}^{Q}\cdot N_{r,j}^{s,Q}+(\Delta b_{r,L_{j}}^{Q}+\Delta b_{L_{j}}^{s,Q})-(d_{r,{\mathrm{IF}}_{12}}^{Q}+d_{{\mathrm{IF}}_{1j}}^{s,Q})+\gamma_{1j}^{Q}\cdot \beta_{12}\cdot DCB_{r,12}^{Q}\\ \delta g_{r,j}^{s}=\gamma_{1j}^{Q}\cdot \beta_{12}\cdot (\delta DPB_{r,12}^{Q}+\delta DPB_{12}^{s,Q})-(\delta b_{r,{\mathrm{IF}}_{12}}^{Q}+\delta b_{{\mathrm{IF}}_{12}}^{s,Q})\\ IFB_{r,j}^{Q}=\left\{\begin{array}{l} 0,j<3\\ -\gamma_{1j}^{Q}\cdot \beta_{12}\cdot DCB_{r,12}^{Q}-d_{r,12}^{Q}+d_{r,j}^{Q},j>3 \end{array}\right.\\ IFCB_{r,j}^{s,Q}=\left\{\begin{array}{l} 0,j<3\\ \delta b_{j}^{s,Q}-\delta b_{{\mathrm{IF}}_{12}}^{s,Q}-\gamma_{1j}^{Q}\beta_{12}\cdot \delta DPB_{12}^{s,Q}+\delta b_{r,j}^{Q}-\delta b_{r{,\mathrm{IF}}_{12}}^{Q}-\gamma_{1j}^{Q}\beta_{12}\cdot \delta DPB_{r,12}^{Q},j>3 \end{array}\right. \end{array}\right.$$

By linearizing and rearranging Equations (1) and (2), the following expressions can be obtained:

(12) $$\left\{\begin{array}{c} \Delta {\bar{P}}_{r,j}^{s,Q}=u_{r}^{s,Q}\cdot V_{X}+c\cdot d{\bar{t}}_{r}^{Q}+Mw_{r}^{s,Q}\cdot ZWD_{r}+\gamma_{1j}^{Q}\cdot {\bar{I}}_{r,1}^{s,Q}+\delta g_{r,j}^{s}+IFB_{r,j}^{Q}+\varepsilon_{r,j}^{s,Q}\\ \Delta {\bar{L}}_{r,j}^{s,Q}=u_{r}^{s,Q}\cdot V_{X}+c\cdot d{\bar{t}}_{r}^{Q}+Mw_{r}^{s,Q}\cdot ZWD_{r}-\gamma_{1j}^{Q}\cdot {\bar{I}}_{r,1}^{s,Q}+\lambda_{j}^{Q}\cdot {\bar{N}}_{r,j}^{s,Q}+IFCB_{r,j}^{s,Q}+\xi_{r,j}^{s,Q} \end{array}\right.$$

where $\Delta {\bar{P}}_{r,j}^{s,Q}$ denotes the difference between the pseudorange observation corrected for satellite-side differential code bias and the initial geometric distance $\rho_{r0}^{s,Q}$; $\Delta {\bar{L}}_{r,j}^{s,Q}$ represents the difference between the carrier-phase observation and the initial geometric distance $\rho_{r0}^{s,Q}$; $u_{r}^{s,Q}$ denotes the line-of-sight unit vector from the satellite to the receiver; and $V_{X}$ represents the correction vector of the receiver coordinates in three dimensions. Equation (12) represents the GNSS undifferenced and uncombined PPP observation model. To account for the different time systems of GPS, BDS-3, and Galileo, the receiver clock offset is estimated separately for each GNSS system, as indicated by the GNSS-specific receiver clock parameter $dt_{r}^{Q}$ in the observation model. In this way, inter-system time offsets are absorbed by GNSS-specific receiver clock parameters rather than being forced into a common receiver clock. In addition, the precise orbit, clock, OSB, and ERP products used in this study are taken from the same WUM final product series, which helps maintain consistency among the time references of different correction products.

The corresponding stochastic model is constructed using the satellite elevation-angle-based weighting method [37]:

(13) $$\sigma^{2}=a^{2}+b^{2}/\sin^{2}\;E$$

where *a* and *b* are constants; *E* denotes the satellite elevation angle in radians.

By combining Equations (12) and (13), the GNSS undifferenced and uncombined PPP model can be established, after which an extended Kalman filter (EKF) is employed to optimally estimate the receiver coordinates, receiver clock offset, zenith wet delay (ZWD), ionospheric delay parameters, and float ambiguities in the undifferenced and uncombined PPP model, yielding the float solutions of the estimated parameters as follows:

(14) $$\bar{X}={\left[\begin{array}{cccc} V_{X}+X_{0} & d{\bar{t}}_{r}^{Q} & ZWD_{r} & \begin{array}{cc} {\bar{I}}_{r,1}^{s,Q} & {\bar{N}}_{r,j}^{s,Q} \end{array} \end{array}\right]}^{T}$$

where $X_{0}$ denotes the initial receiver coordinates, which can be obtained through pseudorange-based single point positioning (SPP); and $V_{X}+X_{0}$ represents the three-dimensional receiver coordinate parameters.

### 2.2. Ambiguity Resolution Method for Multi-GNSS and Multi-Frequency Undifferenced and Uncombined PPP

Cascaded ambiguity resolution is adopted for multi-GNSS and multi-frequency undifferenced and uncombined PPP, in which extra-wide-lane (EWL), wide-lane (WL), and narrow-lane (NL) ambiguities are sequentially fixed. According to the undifferenced and uncombined PPP observation equations described above, the ambiguity parameters ${\bar{N}}_{r,j}^{s,Q}$ generally do not possess integer properties because of the effects of hardware delay biases and unmodeled errors [38]. To enable integer ambiguity fixing, observable-specific bias (OSB) products are first used to eliminate the influence of satellite-side hardware delays on the ambiguity parameters [39]. The ambiguity parameters after satellite-side hardware delay correction are expressed as follows:

(15) $${\hat{N}}_{r,j}^{s,Q}={\bar{N}}_{r,j}^{s,Q}-B_{j}^{s,Q}=N_{r,j}^{s,Q}+B_{r,j}^{Q}$$

where ${\hat{N}}_{r,j}^{s,Q}$ denotes the ambiguity parameter corrected for phase delays, while $B_{j}^{s,Q}$ and $B_{r,j}^{Q}$ represent the satellite-side and receiver-side hardware delays, respectively. Subsequently, to eliminate the influence of receiver-side hardware delays, a reference satellite is selected to construct inter-satellite single-differenced ambiguities. After correction for both satellite- and receiver-side phase biases, the ambiguity parameters theoretically recover their integer nature, thereby enabling integer ambiguity fixing. Based on these corrected ambiguity parameters, a series of extra-wide-lane (EWL) ambiguities can be formed according to the frequency characteristics of different GNSS constellations as follows:

(16) $$\left\{\begin{array}{l} {\hat{N}}_{r,EWL_{23}}^{s_{i}s_{f},G}={\hat{N}}_{r,2}^{s_{i}s_{f},G}-{\hat{N}}_{r,3}^{s_{i}s_{f},G}\\ {\hat{N}}_{r,EWL_{23}}^{s_{i}s_{f},E}={\hat{N}}_{r,2}^{s_{i}s_{f},E}-{\hat{N}}_{r,3}^{s_{i}s_{f},E}\\ {\hat{N}}_{r,EWL_{24}}^{s_{i}s_{f},E}={\hat{N}}_{r,2}^{s_{i}s_{f},E}-{\hat{N}}_{r,4}^{s_{i}s_{f},E}\\ {\hat{N}}_{r,EWL_{25}}^{s_{i}s_{f},E}={\hat{N}}_{r,2}^{s_{i}s_{f},E}-{\hat{N}}_{r,5}^{s_{i}s_{f},E}\\ {\hat{N}}_{r,EWL_{23}}^{s_{i}s_{f},C}={\hat{N}}_{r,2}^{s_{i}s_{f},C}-{\hat{N}}_{r,3}^{s_{i}s_{f},C}\\ {\hat{N}}_{r,EWL_{24}}^{s_{i}s_{f},C}={\hat{N}}_{r,2}^{s_{i}s_{f},C}-{\hat{N}}_{r,4}^{s_{i}s_{f},C}\\ {\hat{N}}_{r,EWL_{25}}^{s_{i}s_{f},C}={\hat{N}}_{r,2}^{s_{i}s_{f},C}-{\hat{N}}_{r,5}^{s_{i}s_{f},C} \end{array}\right.$$

where ${\hat{N}}_{r,EWL_{23}}^{s_{i}s_{f},Q}$ denotes the single-differenced extra-wide-lane (SD EWL) ambiguity, and superscript $s_{i}s_{f}$ represents single-differencing between satellites. After the SD EWL ambiguities are obtained, those with residuals smaller than 0.25 cycle are fixed to integers for each frequency combination using the LAMBDA search method. However, as the ambiguity dimensionality increases, the ratio-test values obtained by the LAMBDA method tend to approach 1. In addition, the long effective wavelength of EWL ambiguities enables instantaneous integer fixing. Therefore, the ratio-test threshold for EWL ambiguity fixing is set to 1.

Once the SD EWL ambiguities are fixed to integers, they can be treated as known values and introduced as virtual observations to constrain the float ambiguity parameters. This reduces the correlation between the ambiguity parameters and other estimated parameters, thereby improving the precision of the float ambiguity estimates. Therefore, the SD EWL ambiguities from all frequency combinations are jointly introduced as virtual observations to constrain the float ambiguity parameters. The corresponding SD EWL ambiguity constraint equation is expressed as follows:

(17) $$\left\{\begin{array}{c} \Delta {\tilde{N}}_{r,EWL_{23}}^{s_{1}s_{f},Q}={\tilde{N}}_{r,EWL_{23}}^{s_{1}s_{f},Q}-{\tilde{N}}_{r,EWL_{23}}^{s_{1}s_{f},Q}\\ \ldots \\ \Delta {\tilde{N}}_{r,EWL_{23}}^{s_{i}s_{f},Q}={\tilde{N}}_{r,EWL_{23}}^{s_{i}s_{f},Q}-{\tilde{N}}_{r,EWL_{23}}^{s_{i}s_{f},Q}\\ \Delta {\tilde{N}}_{r,EWL_{24}}^{s_{1}s_{f},Q}={\tilde{N}}_{r,EWL_{24}}^{s_{1}s_{f},Q}-{\tilde{N}}_{r,EWL_{24}}^{s_{1}s_{f},Q}\\ \ldots \\ \Delta {\tilde{N}}_{r,EWL_{24}}^{s_{i}s_{f},Q}={\tilde{N}}_{r,EWL_{24}}^{s_{i}s_{f},Q}-{\tilde{N}}_{r,EWL_{24}}^{s_{i}s_{f},Q}\\ \Delta {\tilde{N}}_{r,EWL_{25}}^{s_{1}s_{f},Q}={\tilde{N}}_{r,EWL_{25}}^{s_{1}s_{f},Q}-{\tilde{N}}_{r,EWL_{25}}^{s_{1}s_{f},Q}\\ \ldots \\ \Delta {\tilde{N}}_{r,EWL_{25}}^{s_{i}s_{f},Q}={\tilde{N}}_{r,EWL_{25}}^{s_{i}s_{f},Q}-{\tilde{N}}_{r,EWL_{25}}^{s_{i}s_{f},Q} \end{array}\right.$$

where ${\tilde{N}}_{r,EWL_{2j}}^{s_{1}s_{f},Q}$ denotes the fixed SD EWL ambiguity; subscript j represents the frequency index (j=3,4,5); and $\Delta {\tilde{N}}_{r,EWL_{23}}^{s_{1}s_{f},Q}$ denotes the residual of the SD EWL ambiguity. After the fixed SD EWL ambiguities are introduced as constraints on the float ambiguity parameters, the precision of the float solutions can be further improved, thereby accelerating the subsequent WL and NL ambiguity resolution processes. Subsequently, the float ambiguity parameters constrained by the fixed SD EWL ambiguities are combined to form inter-satellite single-differenced wide-lane (SD WL) ambiguities as follows:

(18) $${\hat{N}}_{r,WL_{12}}^{s_{i}s_{f},Q}={\hat{N}}_{r,1}^{s_{i}s_{f},Q}-{\hat{N}}_{r,2}^{s_{i}s_{f},Q}$$

where ${\hat{N}}_{r,WL_{12}}^{s_{i}s_{f},Q}$ denotes the single-differenced wide-lane (SD WL) ambiguity. Ambiguities with residuals smaller than 0.25 cycle are fixed to integers using the LAMBDA search method, with the ratio-test threshold set to 1.5. Meanwhile, partial ambiguity resolution (PAR) is employed for ambiguity subset selection to improve the reliability of WL ambiguity fixing. Similarly, the fixed SD WL ambiguities are introduced as constraints to further improve the precision of the float parameters and accelerate the subsequent NL ambiguity resolution process. The corresponding SD WL ambiguity constraint equation is expressed as follows:

(19) $$\left\{\begin{array}{c} \Delta {\tilde{N}}_{r,WL_{23}}^{s_{1}s_{f},Q}={\tilde{N}}_{r,WL_{23}}^{s_{1}s_{f},Q}-{\tilde{N}}_{r,WL_{23}}^{s_{1}s_{f},Q}\\ \ldots \\ \Delta {\tilde{N}}_{r,WL_{23}}^{s_{i}s_{f},Q}={\tilde{N}}_{r,WL_{23}}^{s_{1}s_{f},Q}-{\tilde{N}}_{r,WL_{23}}^{s_{1}s_{f},Q} \end{array}\right.$$

where ${\tilde{N}}_{r,WL_{23}}^{s_{1}s_{f},Q}$ denotes the fixed SD WL ambiguity; and $\Delta {\tilde{N}}_{r,WL_{23}}^{s_{1}s_{f},Q}$ represents the residual of the SD WL ambiguity. After the fixed SD WL ambiguities are imposed as constraints on the float parameters, the inter-satellite single-differenced ambiguities at the first and second frequencies are combined to form the inter-satellite single-differenced ionosphere-free (SD IF) ambiguity. The SD narrow-lane (SD NL) ambiguity is then derived from the SD IF ambiguity. The resulting SD IF ambiguity and the derived SD NL ambiguity are expressed as follows:

(20) $$\left\{\begin{array}{l} {\hat{N}}_{r,IF_{12}}^{s_{i}s_{f},Q}=\alpha_{12}{\hat{N}}_{r,1}^{s_{i}s_{f},Q}+\beta_{12}{\hat{N}}_{r,2}^{s_{i}s_{f},Q}\\ {\hat{N}}_{r,NL}^{s_{i}s_{f},Q}=\left(\lambda_{IF}{\hat{N}}_{r,IF_{12}}^{s_{i}s_{f},Q}-\frac{\lambda_{j}^{Q}}{\gamma_{12}^{Q}-1}\cdot {\tilde{N}}_{r,WL_{12}}^{s_{i}s_{f},Q}\right)/\lambda_{NL} \end{array}\right.$$

For the derived SD NL ambiguities, the LAMBDA search strategy is employed to obtain the integer solutions, and the PAR strategy is used to identify the optimal ambiguity subset. In this process, the NL residual threshold is set to 0.15 cycle, and the ratio-test threshold is set to 2. After the SD NL ambiguities are fixed, they are introduced as constraints to further update the parameters constrained by the fixed SD WL ambiguities, ultimately yielding the NL ambiguity-fixed solution.

### 2.3. Implementation of the MPAR Algorithm

**Figure 2:**
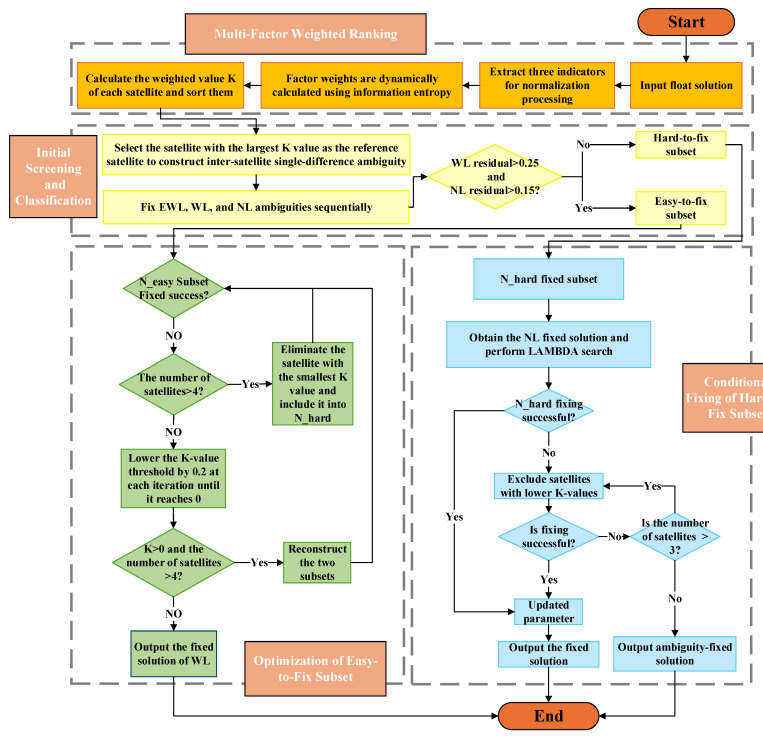
Flowchart of the MPAR Algorithm.

Reliable ambiguity subset selection requires effective characterization of both observation quality and ambiguity estimation quality. Among commonly used observation-quality indicators, the signal-to-noise ratio (SNR) describes the relationship between signal strength and noise level and provides a direct measure of observation quality [40]. After the PPP float solution is obtained, carrier-phase residuals can reflect the consistency of carrier-phase observations, while ambiguity variances indicate the precision of float ambiguity estimates. Therefore, SNR, ambiguity variance, and carrier-phase residuals are jointly adopted in this study to evaluate candidate ambiguities and support adaptive ambiguity subset selection. The implementation flow of the proposed MPAR algorithm is shown in Figure 2.

The MPAR algorithm is implemented through the following steps:

1. Three indicators are first extracted, namely the signal-to-noise ratio (SNR), ambiguity variance ($N_{\mathrm{var}}$), and carrier-phase residual ($L_{res}$). The min–max normalization method given in Equation (21) is then applied to transform these indicators into dimensionless quantities.
  (21) $$\hat{X}=\left\{\begin{array}{l} \frac{X-X_{MIN}}{X_{MAX}-X_{MIN}}\;\;\;for\;\;\;SNR\\ \frac{X_{MAX}-X}{X_{MAX}-X_{MIN}}\;\;\;for\;\;\;N_{\mathrm{var}}\;\;\;or\;\;\;L_{res} \end{array}\right.$$
  where X denotes the current value of SNR, $N_{\mathrm{var}}$, or $L_{res}$. The subscripts MAX and MIN represent the maximum and minimum values, respectively, and $\hat{X}$ denotes the normalized factor.
2. The weighted value of each satellite is calculated, and the satellites are then ranked accordingly. The normalized factors $\hat{X}$ are denoted as $O_{SNR}$, $O_{\mathrm{var}}$ and $O_{res}$, respectively, and their corresponding weight factors are denoted as $W_{1}$, $W_{2}$ and $W_{3}$. The weight factors are determined using the information entropy method, so that the weights can be dynamically adjusted according to the observation environment. First, for each factor, the proportion corresponding to satellite i is calculated as
  (22) $$p^{i}=\frac{O^{i}}{\sum_{i=1}^{n}O^{i}}$$
  where superscript i denotes the satellite. After the proportions are obtained, the information entropy of each factor is calculated as
  (23) $$E=-\frac{1}{\ln n}\sum_{i=1}^{n}p^{i}\ln p^{i}$$
  the normalized difference coefficient is then calculated as
  (24) d=1−E
  which reflects the discrimination capability of each factor. The weight of each factor is subsequently obtained as
  (25) $$W_{j}=\frac{d_{j}}{\sum_{k=1}^{3}d_{k}}$$
  After the weight factors are determined, the weighted value of each satellite is calculated as
  (26) $$K=W_{1}O_{SNR}+W_{2}O_{\mathrm{var}}+W_{3}O_{res}$$
  where
  (27) $$W_{1}+W_{2}+W_{3}=1$$
  Finally, the satellites are ranked according to their calculated K-values, and the satellite with the largest K-value is selected as the reference satellite.
3. In the initial screening stage, the ambiguities are divided into an easy-to-fix subset (N_easy_) and a hard-to-fix subset (N_hard_). After the reference satellite is selected, inter-satellite single-differenced ambiguities are constructed according to the method described in Section 2.2, followed by stepwise fixing of EWL, WL, and NL ambiguities. During WL and NL ambiguity fixing, satellites are first screened based on ambiguity residual thresholds. Satellites whose SD WL ambiguity residuals exceed 0.25 cycle or whose SD NL ambiguity residuals exceed 0.15 cycle are assigned to the hard-to-fix subset N_hard_, while the remaining satellites are assigned to the easy-to-fix subset N_easy_. A full ambiguity fixing attempt is then performed for the N_easy_ subset. If the fixing is successful, the procedure is completed; otherwise, Step (4) is performed. It should be noted that this residual-threshold-based satellite screening step is necessary and must be performed regardless of whether partial ambiguity resolution is subsequently applied.
4. Secondary screening and fixing are performed for the N_easy_ subset. After the full ambiguity fixing attempt fails, secondary screening is conducted by assigning satellites in N_easy_ with K-values smaller than 0.4 to the N_hard_ subset. This step aims to retain satellites with higher observation quality and ambiguities that are easier to fix in the N_easy_ subset. After secondary screening, the LAMBDA search is performed again for the N_easy_ subset. If ambiguity fixing still fails, satellites are removed one by one from N_easy_ in ascending order of their K-values, and each removed satellite is simultaneously assigned to N_hard_. Ambiguity fixing is then attempted again after each removal until the number of satellites in N_easy_ becomes fewer than four. If the number of satellites in N_easy_ is fewer than four and ambiguity fixing remains unsuccessful, the K-value threshold is gradually reduced by 0.2 at each iteration. The N_easy_ and N_hard_ subsets are then reconstructed, and ambiguity fixing is repeatedly attempted until the threshold decreases to K = 0. If the N_easy_ subset is successfully fixed during this process, Step (5) is performed. If fewer than four satellites remain in N_easy_ when K = 0, the PPP WL ambiguity-fixed solution is finally output.
5. Ambiguity fixing is then performed for the N_hard_ subset. After the N_easy_ subset is successfully fixed, the parameters are first updated to obtain the NL ambiguity-fixed solution. The LAMBDA search is then re-executed for the updated N_hard_ ambiguity subset. If the search is successful, the parameters are updated again. If the search fails, satellites are sequentially removed in ascending order of their K-values to progressively achieve partial ambiguity fixing. If ambiguity fixing succeeds during this process, the fixed N_hard_ subset is used again to update the parameters, thereby completing the ambiguity fixing procedure.

## 3. Experiments

### 3.1. Experimental Data

**Figure 3:**
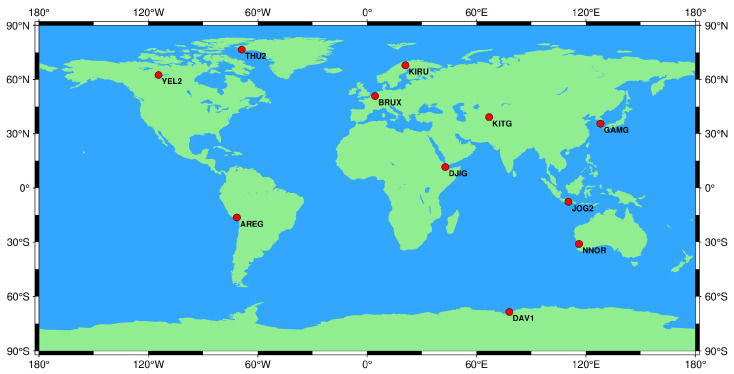
Geographical Distribution of the 11 MGEX Stations Used in This Study.

To comprehensively evaluate the ambiguity-fixing and positioning performance of the proposed MPAR algorithm, 24 h continuous observations collected from 11 globally distributed MGEX stations on day 350 of 2025 were processed and analyzed. The sampling interval of the observation data was 30 s. The geographical distribution of the selected stations is shown in Figure 3.

### 3.2. Experimental Scheme

Three experimental schemes with different frequency configurations were designed for the combined BDS-3, GPS, and Galileo constellations. To assess the effectiveness of different ambiguity subset selection strategies, four ambiguity resolution algorithms were implemented under the undifferenced and uncombined PPP model: full ambiguity resolution (FAR), elevation-angle-factor-based partial ambiguity resolution (ELE), variance-factor-based partial ambiguity resolution (VAR), and the proposed multi-factor ranking and screening partial ambiguity resolution (MPAR) algorithm. For each scheme, ambiguity-fixed PPP solutions were obtained using these algorithms, enabling a systematic comparison of their ambiguity-fixing and positioning performance. In this study, FAR is used as the baseline full ambiguity resolution strategy, in which the reference satellite is selected using an elevation-angle-based K-value and all candidate ambiguities are jointly attempted for fixing; no subsequent K-value-based iterative exclusion is performed after an initial fixing failure. In contrast, ELE and VAR conduct ambiguity subset selection through iterative exclusion based on elevation-angle-based and ambiguity-variance-based K-values, respectively. The proposed MPAR algorithm further improves this subset selection strategy by constructing an entropy-weighted K-value from signal-to-noise ratio, ambiguity variance, and carrier-phase residuals to identify more reliable ambiguity subsets.

Scheme A: For the three constellations, BDS-3 uses five frequencies, namely B1I, B3I, B2a, B1C, and B2b; GPS uses three frequencies, namely L1, L2, and L5; and Galileo uses five frequencies, namely E1, E5a, E5b, E6, and E5ab.

Scheme B: BDS-3 uses four frequencies, namely B1I, B3I, B2a, and B1C; GPS continues to use L1, L2, and L5; and Galileo uses four frequencies, namely E1, E5a, E5b, and E6.

Scheme C: A triple-frequency configuration is used for the BDS-3, GPS, and Galileo constellations. BDS-3 uses B1I, B3I, and B2a; GPS uses L1, L2, and L5; and Galileo uses E1, E5a, and E5b.

To quantitatively assess the positioning accuracy of each scheme, the known coordinates of the corresponding stations obtained from the SINEX (SNX) coordinate file released by the International GNSS Service (IGS) for day 350 of 2025 were used as reference values. Positioning biases in the east (E), north (N), and up (U) directions were then calculated for each scheme. A comprehensive comparison of the four ambiguity resolution algorithms under different frequency configurations was conducted to evaluate the advantages and applicability of the MPAR algorithm in multi-GNSS and multi-frequency PPP ambiguity resolution. The corresponding data processing strategies are summarized in Table 1.

## 4. Results and Discussion

### 4.1. Station-Based Results Analysis

#### 4.1.1. Observation Conditions and Ambiguity-Fixing Rate Analysis

**Figure 4:**
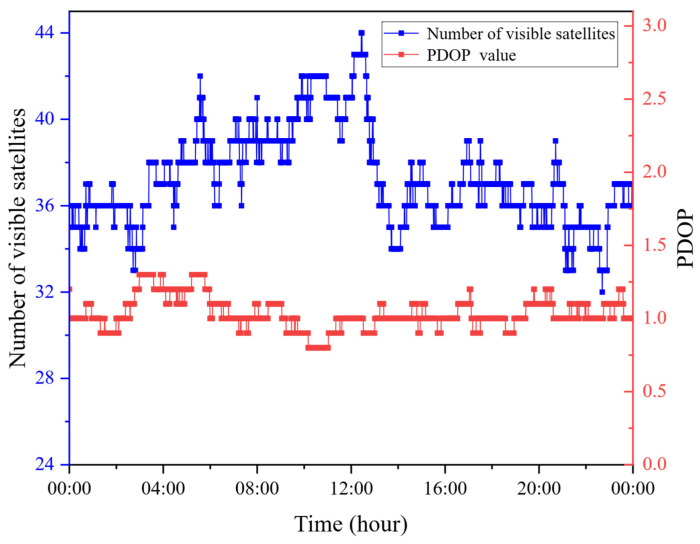
Time Series of Visible Satellites and PDOP Values at the DJIG Station.

The DJIG station was selected as a representative case for station-specific analysis. Using 24 h continuous observations collected at DJIG on day 350 of 2025, PPP performance was evaluated under the experimental schemes described above. Figure 4 illustrates the time series of the number of visible satellites and position dilution of precision (PDOP) values for the combined BDS-3/GPS/Galileo constellation. As shown in Figure 4, the number of visible satellites ranged from 32 to 44, with an average of 37.3, while the PDOP values ranged from 0.8 to 1.3, with an average of 1.0. These results indicate favorable satellite visibility and observation geometry, providing suitable conditions for evaluating multi-GNSS and multi-frequency undifferenced and uncombined PPP. Table 2 illustrates the ambiguity-fixing rates of the different ambiguity resolution algorithms at DJIG under the three experimental schemes. The MPAR and ELE algorithms achieved comparable ambiguity-fixing rates across all schemes, both outperforming VAR, whereas FAR exhibited the lowest fixing rates.

#### 4.1.2. Factor Weight Variation Analysis

**Figure 5:**
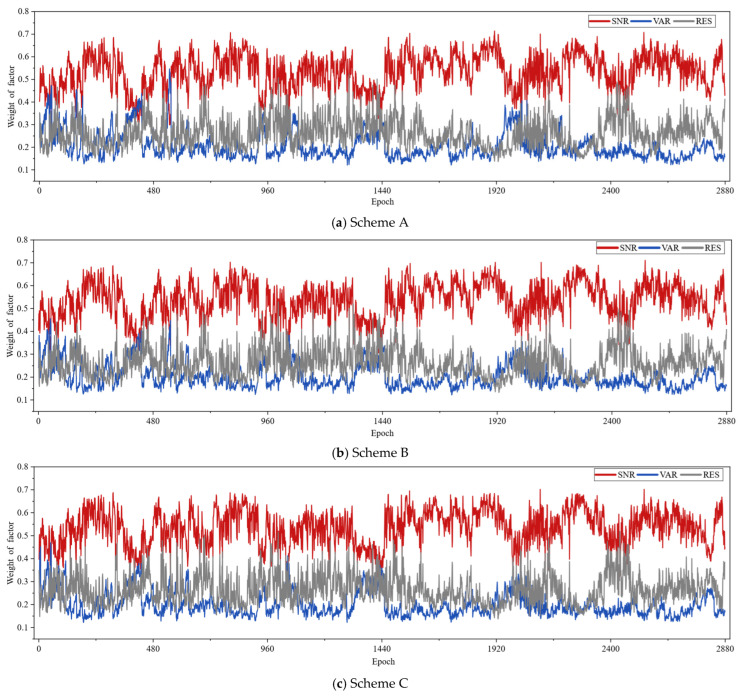
Temporal Variation in Factor Weights.

Figure 5 illustrates how the weights of the three factors vary during the processing of the DJIG station data under the three schemes.

Figure 5 illustrates how the three entropy-weighted factors vary during the processing of the DJIG station data under the three schemes. The results show that the factor weights are not fixed but are adjusted adaptively throughout the solution process. In the entropy-based weighting scheme, the weight assigned to each factor depends on the dispersion of the corresponding normalized indicator among satellites. When an indicator takes similar values for all satellites, it has a relatively high entropy and a small difference coefficient, implying that it provides limited information for distinguishing the reliability of the ambiguities. Accordingly, this factor is assigned a lower weight. In contrast, when an indicator varies substantially among satellites, its entropy decreases and its difference coefficient increases, indicating a stronger ability to discriminate among ambiguities and therefore leading to a higher weight. For example, noticeable fluctuations in the weights of the three factors occur between epochs 1200 and 1440. During this interval, the observation conditions gradually became more favorable, and most of the satellite signals received at the station showed relatively high SNRs. As the SNR values became less discriminative, the weight of the SNR factor decreased, whereas the weights of the VAR and RES factors increased to varying degrees. The three panels also show that the weight variations differ among the three processing schemes. This confirms that the proposed MPAR algorithm can dynamically adjust the weights of the three factors according to the data characteristics.

#### 4.1.3. Ambiguity Residual Accuracy Analysis

**Figure 6:**
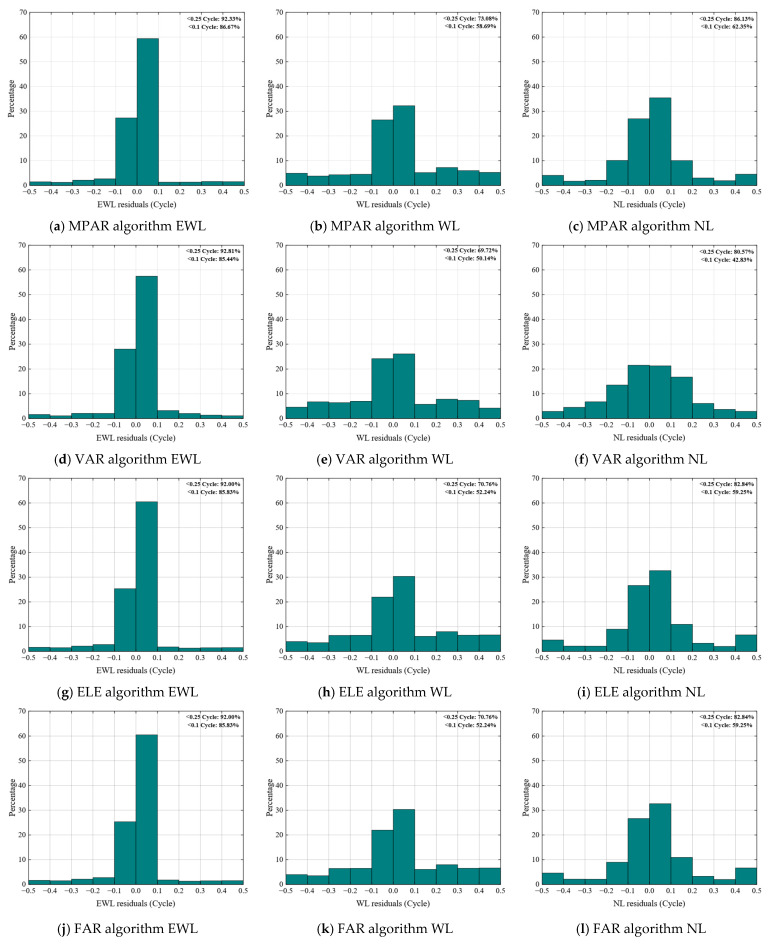
Distribution of Ambiguity Residuals for Different Algorithms under Scheme A.

**Figure 7:**
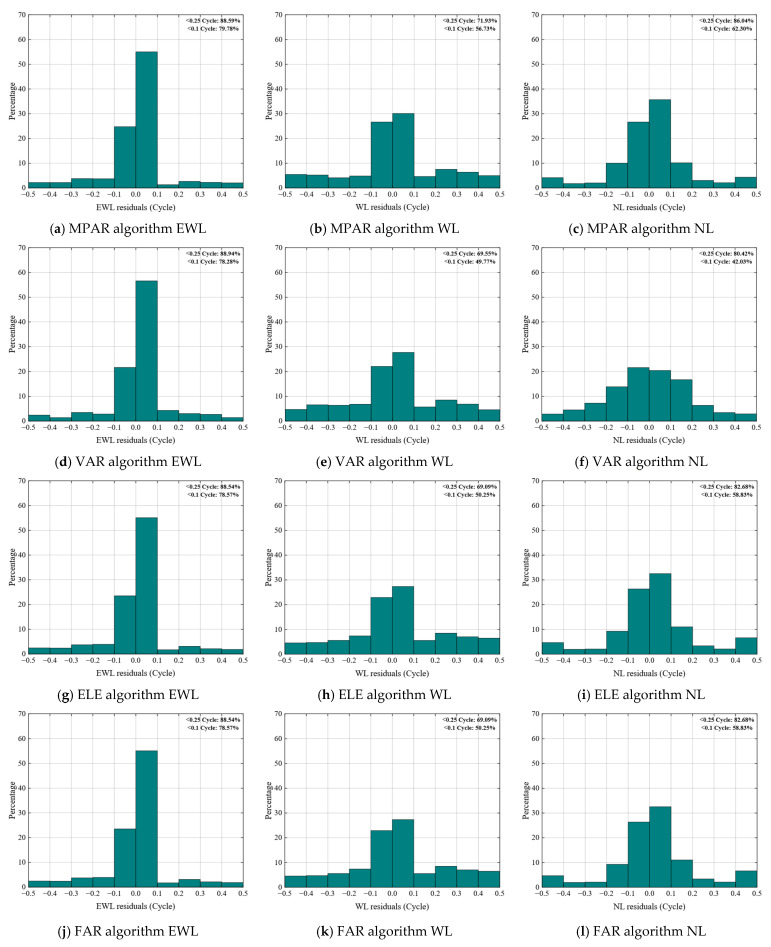
Distribution of Ambiguity Residuals for Different Algorithms under Scheme B.

**Figure 8:**
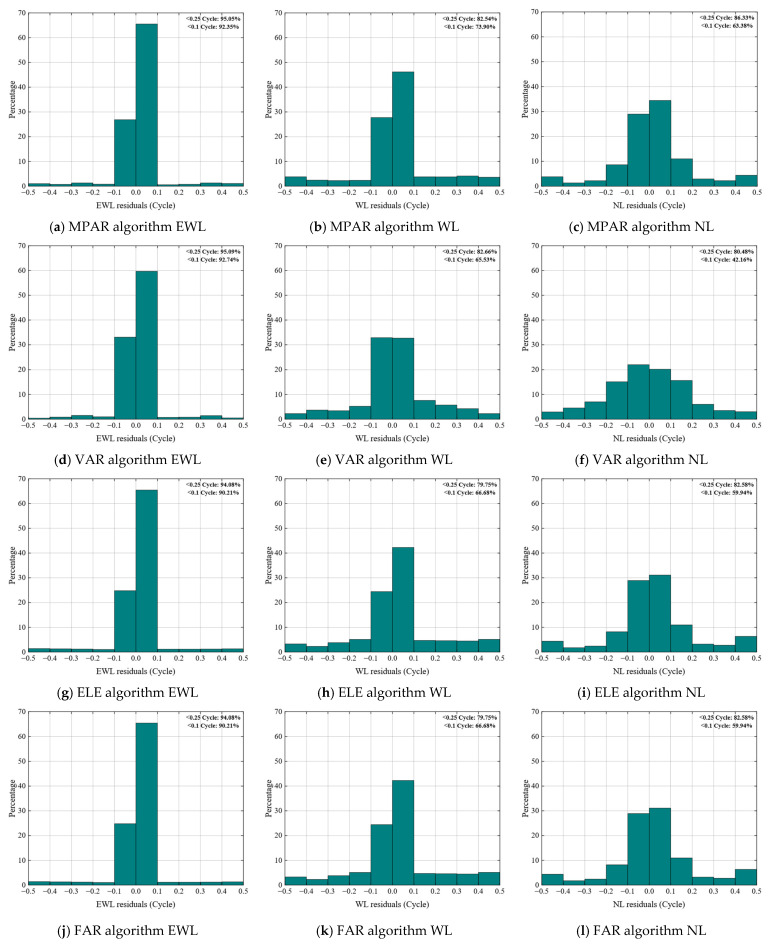
Distribution of Ambiguity Residuals for Different Algorithms under Scheme C.

Figure 6, Figure 7 and Figure 8 present the residual distributions of extra-wide-lane (EWL), wide-lane (WL), and narrow-lane (NL) ambiguities obtained using the four ambiguity resolution algorithms under the three experimental schemes at the DJIG station. The proportions of EWL residuals within ±0.25 and ±0.1 cycle are comparable among the four algorithms. This is mainly because EWL ambiguities have the longest effective wavelengths and are therefore less affected by measurement noise and multipath effects.

Figure 6 illustrates the ambiguity residual distributions under Scheme A. For WL ambiguities, the proportion of MPAR residuals within ±0.1 cycle reaches 58.69%, corresponding to improvements of 17.1%, 12.3%, and 12.3% over VAR, ELE, and FAR, respectively. For NL ambiguities, the corresponding proportion reaches 62.35%, with improvements of 45.6%, 5.2%, and 5.2% over VAR, ELE, and FAR, respectively. These results indicate that the proposed MPAR algorithm improves the concentration of WL and NL ambiguity residuals, particularly when compared with the variance-factor-based subset selection strategy.

The residual distributions of FAR and ELE are nearly identical because both strategies use the elevation-angle-based K-value for reference-satellite selection. However, ELE performs K-value-based sequential exclusion after an initial fixing failure, whereas FAR does not. Since the residual statistics are computed only from successfully fixed ambiguities, the fixed subsets obtained by FAR are generally consistent with those obtained by ELE in successful epochs. Therefore, the similar residual distributions of FAR and ELE mainly result from their common reference-satellite selection strategy and the successfully fixed ambiguity samples used in the residual statistics.

Figure 7 illustrates the ambiguity residual distributions under Scheme B. For WL ambiguities, the proportion of MPAR residuals within ±0.1 cycle reaches 56.73%, representing improvements of 14.0%, 12.9%, and 12.9% over VAR, ELE, and FAR, respectively. For NL ambiguities, the corresponding proportion reaches 62.30%, with improvements of 48.2%, 5.9%, and 5.9% over VAR, ELE, and FAR, respectively. These results further demonstrate that MPAR improves the concentration of WL and NL ambiguity residuals under the four-frequency configuration.

Figure 8 illustrates the ambiguity residual distributions under Scheme C. For WL ambiguities, the proportion of MPAR residuals within ±0.1 cycle reaches 73.90%, representing improvements of 12.8%, 10.8%, and 10.8% over VAR, ELE, and FAR, respectively. For NL ambiguities, the corresponding proportion reaches 62.38%, with improvements of 50.3%, 5.7%, and 5.7% over VAR, ELE, and FAR, respectively. These results indicate that MPAR maintains superior residual concentration under the three-frequency configuration, particularly for NL ambiguity residuals.

The ambiguity residual distributions demonstrate that the MPAR algorithm effectively improves the estimation quality of both WL and NL ambiguities. This improvement is mainly attributed to the proposed multi-factor ranking and screening strategy, which prioritizes high-quality ambiguity subsets for integer fixing and thereby enhances ambiguity-fixing reliability.

#### 4.1.4. Convergence Time and Positioning Accuracy Analysis

**Figure 9:**
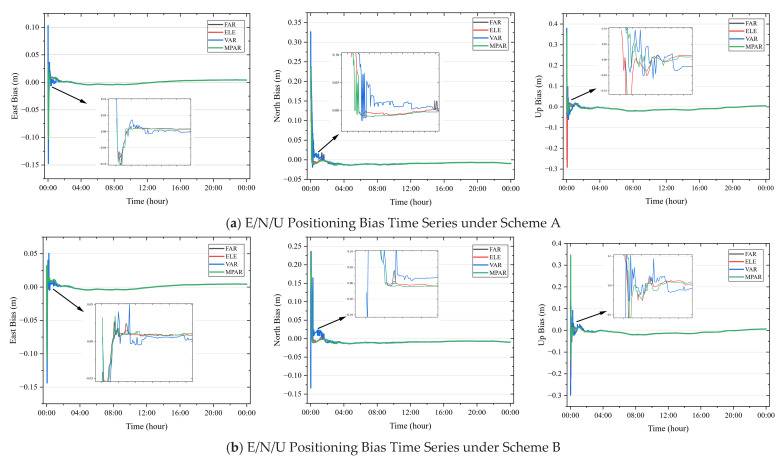

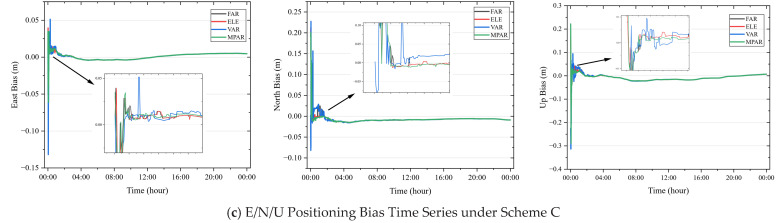
E/N/U Bias Time Series of Different Algorithms under Three Experimental Schemes.

Figure 9 illustrates the east, north, and up (E/N/U) positioning bias time series obtained using the different algorithms under the three experimental schemes. Table 3 lists the convergence times, which are defined as the first epochs from which the coordinate biases in the local east-north-up (ENU) frame remain within 0.1 m for 20 consecutive epochs [41]. Table 4 summarizes the post-convergence horizontal root mean square (RMS) and three-dimensional position RMS values. As shown in Figure 9 and Table 3 and Table 4, MPAR, ELE, and FAR achieve rapid horizontal convergence under all three schemes, with MPAR under Scheme A exhibiting the best performance. By contrast, VAR requires the longest convergence time, although its convergence time decreases as the number of frequencies increases. In the vertical direction, all four algorithms converge rapidly under the three schemes, with MPAR under Scheme A again achieving the best performance. This improvement is mainly attributed to the ability of MPAR to preferentially select high-quality ambiguity subsets through multi-factor ranking, screening, and adaptive weighting, thereby improving ambiguity-fixing reliability and accelerating PPP convergence. In terms of post-convergence horizontal and three-dimensional positioning accuracy, all four algorithms show comparable performance under the three schemes.

The above analysis shows that the MPAR algorithm improves convergence performance and ambiguity-fixing reliability through reliable ambiguity subset selection, with the best overall performance achieved under Scheme A.

### 4.2. Statistical Results Analysis and Discussion

#### 4.2.1. Scheme A

**Figure 10:**
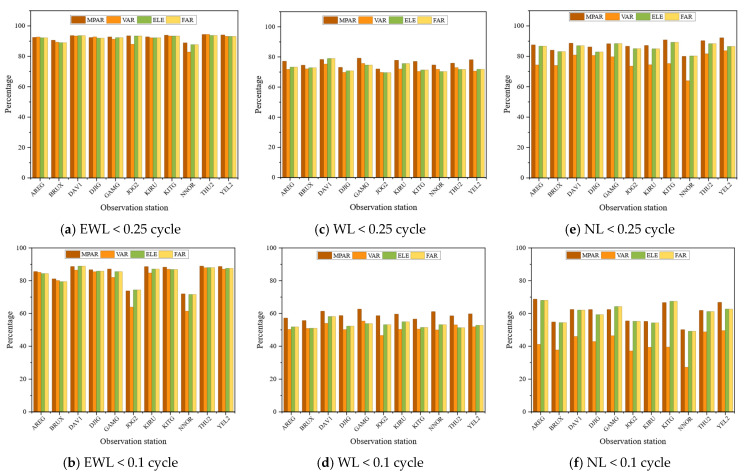
Probability Distributions of Ambiguity Residuals under Scheme A for the 11 Stations.

Figure 10 illustrates the probability distributions of ambiguity residuals after ambiguity resolution under Scheme A for the 11 stations. According to the statistical results, the average proportion of WL ambiguity residuals within ±0.1 cycle reaches 59.05% for MPAR, compared with 51.14%, 53.05%, and 53.05% for VAR, ELE, and FAR, respectively. Thus, MPAR improves the concentration of WL ambiguity residuals within ±0.1 cycle by 15.4%, 11.3%, and 11.3% over VAR, ELE, and FAR, respectively. For NL ambiguities, the average proportion of MPAR residuals within ±0.1 cycle reaches 60.59%, whereas the corresponding proportions for VAR, ELE, and FAR are 41.38%, 59.79%, and 59.79%, respectively. Accordingly, MPAR improves the concentration of NL ambiguity residuals within ±0.1 cycle by 46.4%, 1.3%, and 1.3% over VAR, ELE, and FAR, respectively.

These results indicate that MPAR enables more effective ambiguity subset selection under the five-frequency configuration. Unlike VAR, which relies solely on ambiguity variance, MPAR jointly incorporates SNR, ambiguity variance, and carrier-phase residuals, thereby providing a more comprehensive assessment of ambiguity quality. As a result, ambiguities affected by weak signal strength, large estimation uncertainty, or abnormal carrier-phase residuals can be more appropriately identified and excluded from the fixing subset. Moreover, the entropy-based adaptive weighting strategy dynamically adjusts the contribution of each indicator according to the information distribution of the normalized quality indicators, thereby alleviating the limitations of fixed single-factor ranking. Under Scheme A, the increased number of available frequencies further improves observation redundancy and strengthens the constraints on ambiguity parameters, ultimately enhancing the residual accuracy of both WL and NL ambiguities.

**Figure 11:**
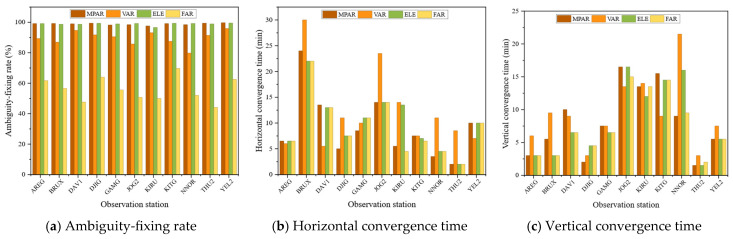
Ambiguity-Fixing Rates and Convergence Times of Different Algorithms under Scheme A.

**Figure 12:**
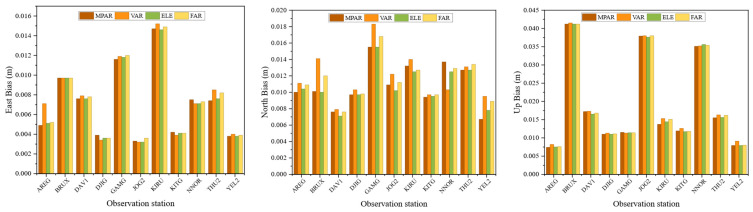
Statistical Results of E/N/U Positioning Biases for Different Algorithms under Scheme A.

Figure 11 and Figure 12 present the statistical results of ambiguity-fixing rates, convergence times, and E/N/U positioning biases for the 11 stations. Table 5 summarizes the average positioning performance metrics of the four algorithms. For ambiguity fixing, the average fixing rates of MPAR, VAR, ELE, and FAR are 98.9%, 89.7%, 98.9%, and 55.9%, respectively. Among the three PAR algorithms, MPAR and ELE exhibit comparable performance, and both outperform VAR. Regarding convergence time, MPAR reduces the horizontal convergence time by 25.0% and 9.9% compared with VAR and ELE, respectively. In the vertical direction, MPAR shortens the convergence time by 13.8% relative to VAR and achieves performance comparable to ELE. MPAR and FAR show comparable horizontal convergence performance, although MPAR has a slightly longer vertical convergence time than FAR. In terms of post-convergence positioning accuracy, all four algorithms achieve comparable results.

These results further demonstrate the effectiveness of MPAR under the five-frequency configuration. The comparable ambiguity-fixing rates achieved by MPAR and ELE indicate that both strategies can effectively identify reliable ambiguity subsets. However, MPAR achieves shorter convergence times than VAR and ELE because it jointly incorporates SNR, ambiguity variance, and carrier-phase residuals, rather than relying on a single ranking factor. This multi-factor subset selection strategy enables more reliable ambiguities to be fixed at an earlier stage, thereby strengthening the constraints on float parameters and accelerating convergence. The slightly weaker vertical convergence performance of MPAR relative to FAR may be associated with the fact that partial ambiguity resolution prioritizes fixing reliability over the number of fixed ambiguities, which may reduce the ambiguity constraints on the vertical component in some epochs. Nevertheless, the comparable post-convergence positioning accuracy among the four algorithms indicates that MPAR improves convergence efficiency without degrading positioning accuracy after convergence.

#### 4.2.2. Scheme B

**Figure 13:**
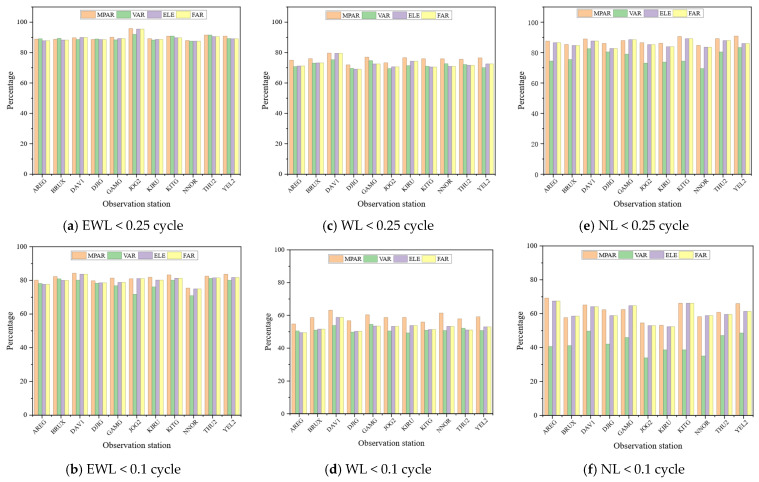
Probability Distributions of Ambiguity Residuals under Scheme B for the 11 Stations.

Figure 13 illustrates the probability distributions of ambiguity residuals after ambiguity resolution under Scheme B for the 11 stations. The average proportion of WL ambiguity residuals within ±0.1 cycle reaches 58.66% for MPAR, representing improvements of 14.5%, 11.4%, and 11.4% over VAR, ELE, and FAR, respectively. For NL ambiguities, the corresponding proportion reaches 61.42%, with improvements of 46.5%, 1.6%, and 1.6% over VAR, ELE, and FAR, respectively. Compared with Scheme A, the WL residual concentration of MPAR decreases slightly, whereas the NL residual concentration remains comparable. These results indicate that MPAR maintains stable ambiguity residual accuracy under the four-frequency configuration. However, its overall performance does not exceed that under Scheme A, suggesting that the richer observation information and stronger ambiguity constraints provided by the five-frequency configuration are more favorable for fully exploiting the advantages of MPAR.

**Figure 14:**
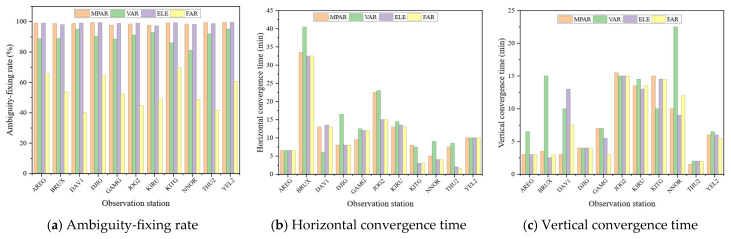
Ambiguity-Fixing Rates and Convergence Times of Different Algorithms under Scheme B.

**Figure 15:**
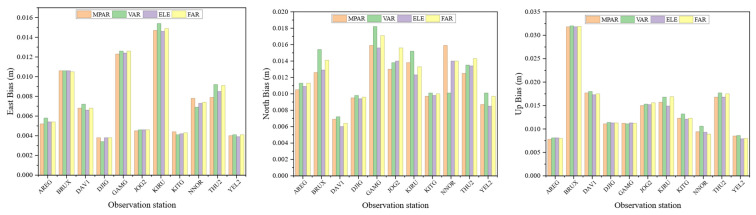
Statistical Results of E/N/U Positioning Biases for Different Algorithms under Scheme B.

Figure 14 and Figure 15 present the statistical results of ambiguity-fixing rates, convergence times, and E/N/U positioning biases for the 11 stations, while Table 6 summarizes the average positioning performance metrics of the four algorithms. The average fixing rates of MPAR, VAR, ELE, and FAR are 98.7%, 89.9%, 98.7%, and 53.7%, respectively. MPAR and ELE exhibit comparable ambiguity-fixing performance, and both outperform VAR and FAR. In terms of convergence time, MPAR reduces the horizontal convergence time by 11.4% compared with VAR, although its horizontal convergence performance is inferior to that of ELE and FAR. In the vertical direction, MPAR shortens the convergence time by 27.2% and 6.3% relative to VAR and ELE, respectively, while achieving performance comparable to FAR. For post-convergence positioning accuracy, all four algorithms achieve comparable results. Compared with Scheme A, the overall convergence advantage of MPAR under Scheme B is less pronounced, particularly in the horizontal component. This suggests that the reduced number of available frequencies weakens observation redundancy and ambiguity constraints, whereas the five-frequency configuration in Scheme A provides more favorable conditions for fully exploiting the advantages of the MPAR algorithm.

#### 4.2.3. Scheme C

**Figure 16:**
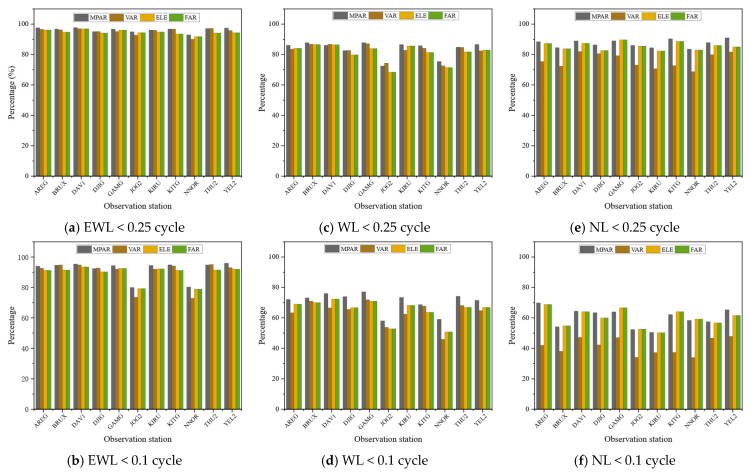
Probability Distributions of Ambiguity Residuals under Scheme C for the 11 Stations.

Figure 16 illustrates the probability distributions of ambiguity residuals after ambiguity resolution under Scheme C for the 11 stations. The average proportion of WL ambiguity residuals within ±0.1 cycle reaches 70.62% for MPAR, representing improvements of 10.9%, 8.2%, and 8.2% over VAR, ELE, and FAR, respectively. For NL ambiguities, the corresponding proportion reaches 60.13%, with improvements of 46.2%, 0.5%, and 0.5% over VAR, ELE, and FAR, respectively. Compared with Schemes A and B, Scheme C shows a higher concentration of WL ambiguity residuals within ±0.1 cycle, mainly because the triple-frequency configuration reduces ambiguity dimensionality and simplifies ambiguity resolution. However, the advantage of MPAR over ELE and FAR becomes less pronounced, particularly for NL ambiguity residuals. This suggests that fewer frequencies reduce the available observation information and weaken the effectiveness of the multi-factor ranking and screening strategy. Therefore, although MPAR remains effective under Scheme C, the five-frequency configuration in Scheme A provides more favorable conditions for fully exploiting the advantages of the proposed MPAR algorithm.

**Figure 17:**
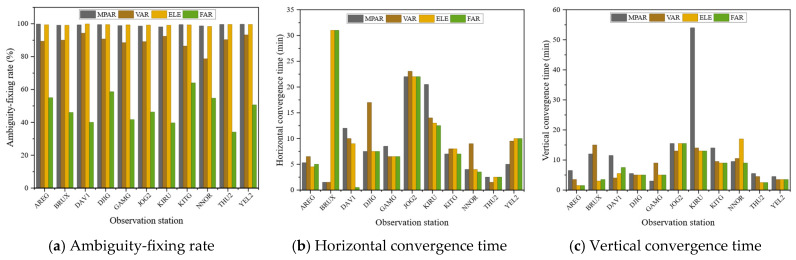
Ambiguity-Fixing Rates and Convergence Times of Different Algorithms under Scheme C.

**Figure 18:**
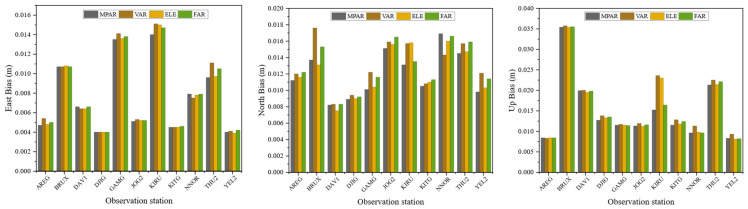
Statistical Results of E/N/U Positioning Biases for Different Algorithms under Scheme C.

Figure 17 and Figure 18 present the statistical results of ambiguity-fixing rates, convergence times, and E/N/U positioning biases for the 11 stations, while Table 7 summarizes the average positioning performance metrics of the four algorithms. The average fixing rates of MPAR, VAR, ELE, and FAR are 99.2%, 89.4%, 99.3%, and 48.2%, respectively. MPAR and ELE exhibit comparable ambiguity-fixing performance, and both outperform VAR and FAR. In terms of convergence time, MPAR reduces the horizontal convergence time by 10.3% and 18.7% compared with VAR and ELE, respectively. However, MPAR requires longer vertical convergence times than VAR and ELE. Compared with FAR, MPAR also shows weaker convergence performance in both the horizontal and vertical directions. For post-convergence positioning accuracy, all four algorithms achieve comparable results. Although MPAR maintains a high ambiguity-fixing rate under Scheme C, its overall convergence advantage is less pronounced than that under Scheme A. This suggests that the triple-frequency configuration reduces ambiguity dimensionality but also limits observation redundancy and ambiguity constraints, particularly for vertical convergence. Therefore, the results under Scheme C further confirm that the five-frequency configuration in Scheme A provides more favorable conditions for fully exploiting the advantages of the MPAR algorithm.

## 5. Conclusions

In this study, an entropy-weighted ambiguity subset selection algorithm based on multi-factor ranking and screening, termed MPAR, was proposed for partial ambiguity resolution in multi-GNSS and multi-frequency undifferenced and uncombined PPP. To evaluate the feasibility and stability of the proposed algorithm, 24 h observations collected from 11 globally distributed MGEX stations tracking BDS-3, GPS, and Galileo on day 350 of 2025 were used. Experimental validation was conducted under five-, four-, and three-frequency configurations, and systematic comparisons were performed with FAR, ELE, and VAR. The main conclusions are summarized as follows:

(1) MPAR achieves average ambiguity-fixing rates of 98.9%, 98.7%, and 99.2% under the five-, four-, and three-frequency configurations, respectively. These values are comparable to those of ELE and higher than those of VAR and FAR, indicating that MPAR maintains stable ambiguity-fixing performance in high-dimensional ambiguity resolution scenarios.
(2) Compared with VAR, MPAR increases the average proportion of WL ambiguity residuals within ±0.1 cycle by 13.6% and improves the corresponding proportion for NL ambiguity residuals by 46.4% on average. This improvement confirms the effectiveness of the proposed two-stage screening mechanism, which combines residual-threshold-based initial screening with K-value-based dynamic subset optimization to improve ambiguity estimation quality.
(3) The advantages of MPAR are most evident under the five-frequency configuration. In this configuration, MPAR achieves the best overall performance, with horizontal and vertical convergence times of 9.1 and 8.1 min, respectively, and an average ambiguity-fixing rate of 98.9%. These results indicate that MPAR can effectively exploit redundant multi-frequency observations and benefit from the proposed entropy-weighted multi-factor ranking and screening strategy for ambiguity subset selection.

Although a full ablation study was not included in the present work, the experimental results still suggest that ambiguity subset selection based on multiple factors is more effective than selection based on a single criterion. Unlike the single-factor VAR and ELE methods, MPAR jointly accounts for SNR, VAR, and RES. Among these indicators, SNR reflects signal strength and the measurement noise level, VAR describes the formal precision of the float ambiguity estimates, and RES indicates the consistency of the observations after parameter estimation. As a result, the proposed ranking score provides a more comprehensive assessment of ambiguity reliability than any single indicator alone. The fact that MPAR consistently outperforms VAR in terms of wide-lane and narrow-lane residual concentration, as well as convergence performance, further indicates that VAR alone is not sufficient to characterize ambiguity quality in high-dimensional multi-frequency PPP scenarios.

In summary, the preliminary experimental results indicate that, by combining multi-factor evaluation with entropy-based adaptive weighting, the MPAR algorithm demonstrates certain advantages in fast and reliable ambiguity fixing under high-dimensional ambiguity scenarios. Its advantages are most evident under the five-frequency configuration, particularly in terms of overall convergence and positioning performance, indicating its suitability for multi-GNSS and multi-frequency PPP applications. Nevertheless, the experimental results also show that, although MPAR achieves horizontal convergence performance comparable to FAR under the five-frequency configuration, its vertical convergence performance still requires further improvement. This may be related to the weaker vertical satellite geometry and the stronger coupling among height, zenith wet delay, residual ionospheric effects, and low-elevation observations. In addition, the experimental validation in this study was limited to a single 24 h dataset, which may constrain the generalizability of the findings. Future work will therefore focus on several aspects. First, systematic ablation experiments will be conducted to quantify the individual contributions of SNR, ambiguity variance, carrier-phase residuals, multi-factor integration, and entropy-based adaptive weighting to ambiguity subset selection. Second, the proposed MPAR algorithm will be compared with more recently developed multi-factor PAR strategies to further examine its advantages and limitations. Third, adaptive thresholds incorporating vertical-direction constraints, multi-factor-based dynamic K-value thresholds, and improved screening rules for hard-to-fix ambiguity subsets with ionospheric prior constraints will be investigated to further improve vertical convergence performance. Finally, multi-day and multi-seasonal datasets collected under different ionospheric activity levels and challenging environments with severe signal blockage will be used to provide a more comprehensive assessment of the robustness and applicability of the proposed algorithm.

## Figures and Tables

**Table 1:** Data Processing Strategies Used in This Study.

Processing Item	Solution
Satellite elevation cutoff angle	10°
Weighting strategy	A priori residual of pseudorange observations: 0.3 m A priori residual of carrier-phase observations: 0.003 m
Satellite orbit and clock products	Corrected using WUM final precise ephemeris and precise clock products
Satellite pseudorange and carrier-phase hardware delays	Corrected using WUM final OSB products
Earth rotation parameters	Corrected using WUM final ERP products
Coordinate parameters	Estimated as white noise
Receiver clock parameters	Estimated as white noise
IFB parameters	Estimated as a random walk for four-frequency and higher observations
Tropospheric delay	Dry delay: Saastamoinen model Wet delay: random walk
Ionospheric delay	Estimated as a random walk
Parameter estimation	EKF

**Table 2:** Ambiguity-Fixing Rates of Different Algorithms at the DJIG Station under Three Experimental Schemes.

	Algorithm	FAR	ELE	VAR	MPAR
Scheme					
Scheme A		64.0%	99.3%	91.7%	99.4%
Scheme B		64.3%	99.3%	90.3%	99.3%
Scheme C		58.6%	99.5%	90.7%	99.5%

**Table 3:** Convergence Times of Different Algorithms under Three Experimental Schemes.

Scheme	Horizontal Convergence Time (min)				Vertical Convergence Time (min)			
	FAR	ELE	VAR	MPAR	FAR	ELE	VAR	MPAR
Scheme A	7.5	7.5	11.0	5	4.5	4.5	3.0	2.0
Scheme B	8.0	8.0	16.5	8.0	4.0	4.0	4.0	4.0
Scheme C	7.5	7.5	17.5	7.5	5.0	5.0	5.0	5.5

**Table 4:** Post-Convergence Positioning Accuracy Statistics of Different Algorithms under Three Experimental Schemes.

Scheme	Horizontal RMS (cm)				Position RMS (cm)			
	FAR	ELE	VAR	MPAR	FAR	ELE	VAR	MPAR
Scheme A	1.05	1.04	1.08	1.05	1.53	1.52	1.56	1.52
Scheme B	1.03	1.02	1.03	1.02	1.53	1.52	1.54	1.51
Scheme C	1.00	0.98	1.03	0.98	1.68	1.65	1.72	1.60

**Table 5:** Average Positioning Performance Metrics of Different Algorithms under Scheme A.

Algorithm	Convergence Time (min)		Fixing Rate (%)	RMS (cm)				
	Horizontal	Vertical		East	North	Up	Horizontal	Position
MPAR	9.1	8.1	98.9	0.7	1.1	1.9	1.3	2.3
VAR	12.2	9.4	89.7	0.7	1.2	2.0	1.4	2.4
ELE	10.1	8.1	98.9	0.7	1.1	1.9	1.3	2.3
FAR	9.2	7.6	55.9	0.7	1.1	1.9	1.3	2.3

**Table 6:** Average Positioning Performance Metrics of Different Algorithms under Scheme B.

Algorithm	Convergence Time (min)		Fixing Rate (%)	RMS (cm)				
	Horizontal	Vertical		East	North	Up	Horizontal	Position
MPAR	12.4	7.5	98.7	0.7	1.2	1.4	1.4	2.0
VAR	14.0	10.3	89.9	0.8	1.2	1.5	1.4	2.1
ELE	10.9	8.0	98.7	0.7	1.2	1.4	1.4	2.0
FAR	10.8	7.5	53.7	0.8	1.2	1.4	1.4	2.0

**Table 7:** Average Positioning Performance Metrics of Different Algorithms under Scheme C.

Algorithm	Convergence Time (min)		Fixing Rate (%)	RMS (cm)				
	Horizontal	Vertical		East	North	Up	Horizontal	Position
MPAR	8.7	12.9	99.2	0.8	1.2	1.5	1.4	2.1
VAR	9.7	8.3	89.4	0.8	1.3	1.6	1.5	2.2
ELE	10.7	7.3	99.3	0.8	1.2	1.6	1.4	2.2
FAR	9.8	6.8	48.2	0.8	1.3	1.5	1.5	2.1

## Data Availability

The data presented in this study are available in the article. Further inquiries can be directed to the corresponding author.
